# Study protocol for the description and evaluation of the “Habit Coach” - a longitudinal multicenter mHealth intervention for healthy habit formation in health care professionals

**DOI:** 10.1186/s12889-022-13986-0

**Published:** 2022-09-04

**Authors:** Anna Vogelsang, Clara Hinrichs, Lena Fleig, Ines Pfeffer

**Affiliations:** 1 Faculty of Humanities, MSH Medical School Hamburg - University of Applied Sciences and Medical University, Am Kaiserkai 1, 20457 Hamburg, Germany; 2 Faculty of Sport Science - Department of eHealth and Sports Analytics, Ruhr-University Bochum, Gesundheitscampus - Nord 10, 44801 Bochum, Germany; 3 Faculty of Natural Sciences- Department of Psychology, Medical School Berlin, Rüdesheimer Straße 50, 14197 Berlin, Germany

**Keywords:** Action planning, Goal setting, Automaticity, Physical activity, Nutrition, Mindfulness

## Abstract

**Background:**

The adoption of a healthy lifestyle plays a crucial role for the health and well-being of health care professionals. Previous e- and mHealth interventions relied on deliberative psychological processes (e.g., intention, planning) to target lifestyle changes, while revealing mixed efficacy. The additional potential of non-deliberative, automatic processes (i.e., habits) for behavior change has been understudied in interventions so far. The Habit Coach mHealth intervention combines deliberative and non-deliberative processes to support health care professionals in forming healthy physical activity, nutrition and mindfulness habits in daily life. The aim of this paper is to outline the study protocol including a detailed description of the mHealth intervention, evaluation plan, and study design. The purpose of this trial is to understand healthy habit formation in health care professionals over time.

**Methods:**

A one-arm, multicenter mHealth intervention study will be conducted. Behavioral and psychosocial predictors will be collected via within-app questionnaires across a 100-day period at baseline, post, as well as at weekly assessments. To understand habit formation across time, linear mixed models will be used.

**Discussion:**

This trial aims to unravel the role of motivational and volitional determinants for healthy habit formation across multiple health behaviors in health care professionals embedded in a mHealth intervention.

**Trial registration:**

This trial is registered in the German Clinical Trials Register, DRKS-ID DRKS00027156. Date of registration 17 November 2021.

**Supplementary Information:**

The online version contains supplementary material available at 10.1186/s12889-022-13986-0.

## Background

The tasks of care giving occupations are stressful and emotionally exhausting [1]. At the same time, their work environment exposes them to various hazards ranging from infectious diseases to radiation [2–4] as well as irregular and long work schedules. While long working hours have been positively associated with lower health, including higher body weight [5] and insufficient physical activity [6], meaningful engagement in health protective and promoting activities (e.g., food preparation, exercising) often comes up short [7]. Consequently, there is a strong rationale and need for fostering health promoting behaviors in health care professionals [8].

Besides the importance of the workplace, health care professionals’ individual health behavior plays a decisive role for their health and well-being [9]. Replacing well-established behavioral patterns with healthier alternatives, however, appears per se as challenging, particularly for health care professionals. Behavioral sciences often apply theories to explain individual behavior change, with a preponderant focus on social cognitive theories ([10]; e.g., the social cognitive theory [11], the theory of planned behavior [12] or the transtheoretical model [13]). These models share the common notion that individuals change their behavior as a result of deliberate psychological processes including self-efficacy and intentions. Meta-analytical evidence on the efficacy of behavioral interventions, however, revealed only short-term behavior changes, and mainly for those interventions that incorporated deliberate, self-regulation techniques such as planning, self-monitoring and feedback provision [14]. As such, while behavioral interventions based on traditional social cognitive models show promising results, there still remains room to expand upon different theoretical angles to change behavior [15, 16].

### Understanding habit formation and its predictors

More recently non-conscious, automatic processes, such as habits, received credit in the behavior change literature [17, 18]. *Habits* can be defined as process by which a stimulus automatically creates an impulse towards action, based on learned stimulus–response associations[19, 20]. The traditional perspective of habit formation implies repetition of an intended behavior in a stable context [21, 22], to the extent that after sufficient repetition, the intended behavior will be elucidated by cues in the environment rather than through an ongoing and conscious decision making process [23].

**Fig. 1:**
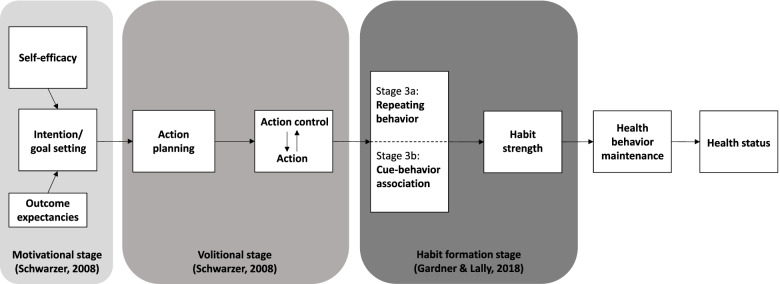
Theoretical framework for the present study based on Schwarzer [25] and Gardner and Lally [26]

In line with these advancements, several attempts have been undertaken to identify strategies that could facilitate and support the formation of healthy habits. Gardner & Lally [24] developed a framework to aid the conceptual organization of antecedents of the habit formation process, proposing the categorization of determinants into stages, much like in well-established stage models of behavior change (e.g., health action process approach, HAPA, [25]). In the first stage, the motivational stage (1), people need to come to a decision to perform a new behavior (Fig. 1). Deliberation over action and potential consequences result in intention formation. In the following volitional stage (2), people attempt to translate their intentions into action. Individuals need to make use of self-regulation strategies to initiate action. In the third stage (3a), people need to repeat the new behavior, which requires sustained motivation and self-regulation skills. At the final stage (3b), the intended behavior is repeated in a way to support the development of habit associations. While stage 3a is per se about behavioral repetition, 3b emphasizes the strengthening of cue-response associations. Together both stages culminate in the formation of a new habit.

Building on this integrated framework, outcome expectancies and self-efficacy beliefs complement the process of intention formation (stage 1). Indeed, before a new behavior becomes automatic, individuals need to set a specific behavioral *goal* (e.g., exercising more, eating healthier). Although the latter initially requires discipline and memory performance it should—over time—become habitual after sufficient repetition of the behavior. Goals have been shown to be important antecedents of action initiation [27]. Individuals most likely form a goal when they anticipate positive consequences as a result of their intended behavior (i.e., outcome expectancies) and feel capable to perform the intended behavior despite barriers (i.e., self-efficacy, [28]). While *outcome expectancies* refer to the perceived physical, affective (self-evaluative) and social consequences of one’s behavior, *self-efficacy* is defined as the ability to control one’s behavior to produce desired outcomes [25]. In terms of intervention strategies, information provision on beneficial consequences of action or letting individuals choose behaviors they feel confident about to execute, set the stage for the formation of habits by establishing a motivational basis [29]. Moreover, research has shown that context-dependent repetition was most likely when individuals formulated goals that were specific, achievable and realistic [30]. In contrast, low quality self-chosen goals that were vaguely defined, specified numerous behaviors, and failed to identify a context for action were suboptimal for habit formation [31, 32].

To foster action initiation (stage 2), self-regulation strategies such as *planning* come into play [33]. Planning helps people to act in favorable situations. By anticipating contexts suitable for behavioral execution in addition to planning how the behavior will be performed in a given context, facilitates developing cue-behavior associations as indicated in stage 3b [24, 34]. As a special form of planning, action planning describes a process in which a person determines specifically when, where and how an intended behavior will be executed. While health care professionals who make use of planning are more likely to engage in health promoting behaviors [35, 36], the underlying processes why and how they build a habit as a result are less clear. Presumably, planning makes a specific cue more easily available in memory so that when exposed to the cue, people are more likely to remember and execute the behavior [27]. As soon as an action plan has been formed, the intended behavior is more likely to be triggered automatically by the contextual cue rather than by a deliberative weighing of pros and cons [37].

Lastly, *self-monitoring* of goals presents an additional facilitative self-regulatory strategy conducive to action control (i.e., the control of self-regulation and voluntary action), where the behavior is continuously monitored and evaluated with respect to a behavioral standard [38]. While self-monitoring has been proven to be a valuable component of behavior change interventions to reduce sedentary behavior [39], its supportive role in the habit formation process (i.e. increasing automaticity of a new health behavior) has been shown across various health behaviors, such as diet and physical activity ([40–42]; for a literature review see [29]).

Some habit formation prerequisites, such as goal setting, action planning and self-monitoring, have been shown to support behavior change (e.g., physical activity [43];), also in worksite interventions [44]. Findings from systematic reviews of traditional worksite based lifestyle interventions, however, indicated mixed findings, showing that 40% to 45% of the included intervention studies did not reveal positive effects on physical activity [45, 46]. Lack of intervention effects was mainly attributed to insufficient theoretical behavioral change rationales of interventions [45] as well as high costs and disruption of the working day [47], with the latter most likely preventing program implementation in the first place [48]. Therefore, there is a need for brief evidence- and theory-based interventions that are feasible to be implemented throughout a busy workday.

Delivering behavioral interventions via electronic health (eHealth) or mobile health (mHealth) technology might overcome aforementioned implementation problems. Still, research investigating the efficacy of digital interventions (i.e., e- and mHealth) presented mixed results: while evidence exists that points to the effectiveness of e- and mHealth interventions in promoting physical activity (for a meta-analysis in adults see Laranjo et al., [49]) and healthy eating in people with non-communicable diseases [50, 51], other reviews report positive – albeit highly variable and often small effects on behaviors such as physical activity, exercise and tobacco use [52, 53]. Mixed mHealth effectiveness was ascribed to poorly described theoretical backgrounds and behavior change techniques, as well as uncertainties about effective dosage and delivery modes [54–56]. Taken together, this lack of clarity explains why researchers frequently start from scratch when developing new intervention programs. Publications of study protocols that precisely describe the active ingredients (i.e., mechanisms of action), dose and mode of the interventions are therefore required [57].

### Objectives

The present study outlines the protocol for a longitudinal, multicenter single-arm study among health care professionals investigating the role of theory-based motivational and volitional predictors for healthy habit formation in daily life based on a mHealth intervention targeting physical activity, healthy nutrition and mindfulness behaviors (i.e., engaging in mindfulness techniques and relaxation exercises). The aim of this paper is to outline the study protocol including a detailed description of the mHealth intervention, the evaluation plan, and the study design. The main purpose of this trial is to understand the habit formation process for physical activity, nutrition and mindfulness behaviors and its predictors. As preliminary hypothesis (hypothesis 1 a-e), we expect to find positive changes from baseline to post assessments (i.e., 100 days post baseline) in: a) habit strength, b) motivational constructs (i.e., intention, outcome expectancies, self-efficacy), c) volitional constructs (i.e., action planning), d) health behavior (i.e., physical activity, nutrition, mindfulness behaviors) and e) health status (i.e., stress, back pain, well-being, physical and psychological health, body mass index). For the primary endpoint (habit strength over time) and to understand predictors of habit formation over time, we hypothesize that a) habit strength at post-assessment is predicted by baseline motivational and volitional constructs, controlling for baseline habit strength (hypothesis 2), b) that health behavior and c) health status at post assessment are predicted by motivational and volitional constructs as well as habit strength at baseline (hypotheses 3 and 4, respectively). Finally, we hypothesize that next-week habit strength will show positive associations with behavior-related motivational (5a) and volitional constructs over time (5b).

Additionally, we conduct a process evaluation by evaluating participants’ experiences and satisfaction with the app. For the process evaluation a mixed-methods approach will be applied involving questionnaires and structured interviews. The items addressed in this protocol paper are based on the 2013 Standard Protocol Items: Recommendations for Interventional Trials (SPIRIT) statement [[58]; see Additional file 1].

## Methods/design

### Sample

The sample consists of employees of different hospitals and care facilities in Germany (blinded for review). Recruitment takes places through the company health care insurances, who have funded the study, as well as other project partners. In case hospitals show interest in the study, the hospital and the workplace health management receive further information on the study. A landing page, specifically developed for each facility and for the purpose of this study, features all study related information and gives interested participants the chance to register for participation (example link to landing page https://aeroscan.com/klinikumkarlsruhe/). The recruitment procedures will continue until the proposed sample size is reached (see Statistical power). Different strategies were applied per hospital for achieving adequate participant enrolment, including an awareness campaign (i.e., fun facts about health and wellness, video clips), posters and flyers (e.g., featuring healthy meal ideas), and a QR-code campaign, for which QR-codes (connected to short exercises and stretches) were displayed at locations at which the staff usually has to wait such as the coffee machine. Additionally, the trial is announced through the hospitals’ intranet (e.g., via email). From a planned in-person onboarding event was foreseen due to the Covid-19 pandemic. To be eligible for participation, participants should be affiliated with a hospital or care facility, should have access to a smartphone or a PC/laptop and should have sufficient knowledge of the German language as well as the ability to comprehend and process the assessment and instruction material. Participants younger than 18 years of age will be excluded from participation. Furthermore, any pre-existing diseases of the musculoskeletal system or diseases such as osteoporosis or arthrosis (based on participant indication), will exclude participants from the trial as well. Participants are not blinded to the intervention.

### Ethical approval

This study has been approved by the Ethic Committee of the (blinded for review; Ethics Committee No.: 2021/136) and has been registered in the German Clinical Trials Register (DRKS-ID: DRKS00027156; AV: 17 November 2021, original, AV: 23 February 2022, amendment 01). The primary reason for the amendment was a refinement of hypotheses. Additional modifications will be reported to the German Clinical Trials Register. Written informed consent from each participant will be obtained and stored on the Aeroscan servers, which are hosted by the Domain Factory in Germany (see Data management plan for more details).

### Study procedure

**Fig. 2:**
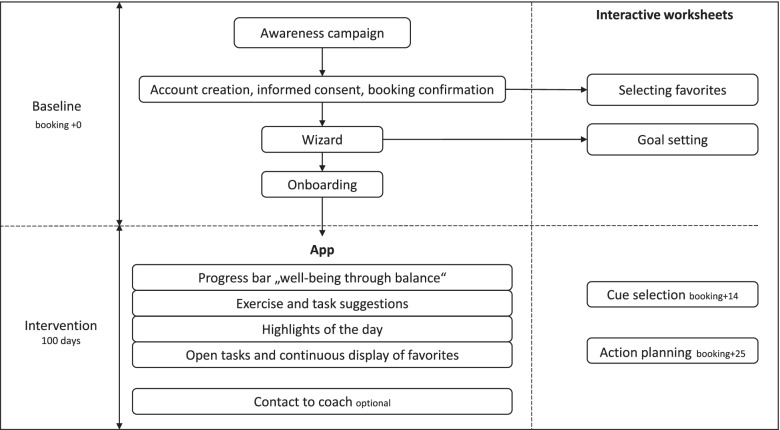
Study flow. Note. Interactive worksheets have been added to the Habit Coach by the team of independent experts in health and exercise psychology

The current protocol focuses exclusively on the evaluation plan of a one-arm, multicenter mHealth interventional study and the post-hoc description of the Habit Coach app (i.e., after the development phase of the app). The trial is undertaken by independent external research experts in health and exercise psychology, presenting an important quality criterion for the project. Figure 2 depicts the study flow. Through the registration process in the app, written informed consent will be obtained from individuals. Upon registration, participants receive a 100-days access to the Habit Coach app. Besides the usual features, the app includes four interactive worksheets to aid the habit formation process, which have been specifically developed for this study by the team of independent experts in health and exercise psychology.

### Description of the app

The app has been developed by the Aeroscan GmbH (limited liability company) and consists of different versions that each fit a specific work context (e.g., home office program, nursing program). The present study exclusively focuses on the nursing program, which is available as a progressive web-app since March 2020. An updated single-page-app is available since November 2021, which will be included in the present study. The app is compatible with desktops and all kinds of mobile devices. The app is accessible only through specific booking codes provided by the Aeroscan company (i.e., not available in common app-stores). A multidisciplinary team consisting of professionals with expertise in nursing, sports and exercise science, physiotherapy, nutrition, stress management and human–computer interactions were involved in the planning and development of the app content and features. The app was devised in iterative processes, involving input from both target group members (i.e., health care professionals) and experts. All within-app exercise and task suggestions were designed to be implemented in daily routines (i.e., within and outside the work context), and in close collaboration with the nurses (i.e., participatory approach) to enhance both personal relevance and acceptance of the app content. Programming of the app was conducted by the Aeroscan IT, being responsible for all tasks related to computer science (i.e., programming of app, surveillance of data servers, etc.).

### Description of the intervention

In order to promote better reporting of interventions, we used the template for intervention description and replication (TIDieR) checklist ([59], [Additional file 2]). The intervention consists of a mobile app (the Habit Coach) with a 100-days access and interactive worksheets, targeting healthy habit formation for the behaviors physical activity, nutrition and mindfulness in health care professionals (Fig. 2). Participants are supported during the entire behavior change process (Fig. 1) to set individual health goals, develop action plans and self-monitor one’s goals in order to facilitate healthy habit formation for multiple health behaviors. The interactive worksheets have been conceptualized by the team of independent experts in health and exercise psychology and added to the regular app-features. The app is location-independent and can be used in and outside the hospital context.

Upon the account creation, participants receive a booking confirmation mail together with the first interactive worksheet called “Selecting favorites” (booking date + 0, [see Additional file 3A]). Through this worksheet, participants are encouraged to select their favorite physical activity, nutrition, and mindfulness exercises and tasks from the within-app catalog within the first couple of weeks and to mark these as favorites (by means of a bookmark symbol, [see Additional file 4A]). Additionally, participants are informed about the importance of repeating selected exercises and tasks in stable contexts as opposed to continuously selecting new exercises. Allowing participants to choose their favorite exercises and tasks enhances their need for autonomy satisfaction and their positive outcome expectancies. Furthermore, by offering participants to choose to incorporate their favorite exercises and tasks in their daily routines (as opposed to only within or outside the work context), reduces negative outcome expectancies (e.g., lack of time). Participants are encouraged to repeat these favorite exercises and tasks on a daily basis over the 100-days period in order to foster cue-behavior associations and therefore habit formation. To ensure that none of the participants misses out on selecting favorite exercises and tasks, a reminder email is sent.

After the account creation, participants will be asked to characterize their job profile, to define their interests in health related topics, to set a personal health goal and to estimate their current physical activity, nutrition and mindfulness behaviors as well as their knowledge with regard to these three behaviors. This so-called wizard (Additional File 4B) is used to tailor the app’s content to participants’ needs, work and private (i.e., family) situation, and personal preferences/interests in health and wellness-related topics (e.g., sleep quality, brain gym, healthy snacks, outdoor activities, cooking with kids). This tailoring will increase the personal relevance of the app content and enhance adherence to the intervention overall. Although the wizard is displayed with the first log-in, participants can make changes to their preferences and interest by filling in the wizard a second or third time during the intervention period. Changes in the wizard (i.e., preferences) will result in changes in offered content but not in changes in self-chosen favorites to prevent disruptions to the habit formation process. In order to facilitate the setting of a personal health goal, participants have the opportunity to download an interactive worksheet (called “Goal setting”, [see Additional file 3B]). This goal setting worksheet provides participants with step by step instructions on how to form “SMART” health goals (i.e., goals that are specific, measurable, achievable, relevant, time bound).

Next, participants are guided through an onboarding process, intended to explain the app’s features and functions. The Habit Coach app is constructed to follow the theme “well-being through balance” for each of the target behavioral domains: physical activity, nutrition and mindfulness. To track and self-monitor balance in all three domains, a progress bar is displayed on the home screen, indicating the average positive and negative progress in each behavioral domain of the last seven days [see Additional file 4A].

The within-app catalogue of physical activity, nutrition, and mindfulness exercises consists of a variety of exercises and tasks differing in duration, intensity and target (e.g., stretching, walking; [see Additional file 4C]). These exercises and tasks are embedded in specific situations/contexts (e.g., during lunch breaks, while preparing medication, during team meetings, on the way home, while playing with kids) encountered by health care professionals throughout the (work)day. It is expected that these everyday situations/contexts will elicit the desired behavior automatically over time after almost daily repetition of that behavior in stable situations/contexts. All physical exercises are explained with a short video, featuring a nurse (dressed as such) engaging in a given exercise. The nurse should function as a role model for participants. Furthermore, exercise and task suggestions consist of a) activity elements intended to motivate participants to take action and b) knowledge elements supposed to motivate participants to learn something new [see Additional file 4D]. Lastly, exercise and task suggestions rotate regularly and rotation depends on participants’ balance progress. As soon as participants engage in an exercise or task, they can tick a box to indicate that they have completed the exercise or task, which provides them with points. Collecting points, as a gamification element, translates into the progress bars and is intended to increase engagement with the intervention. For instance, in case a participant is mainly interested in nutrition and mindfulness tasks during a week, positive progress is made in the areas of nutrition and mindfulness, and negative progress is made for physical activity. To restore balance among all three behaviors, participants will automatically receive more suggestions for physical activity the upcoming week and fewer for nutrition and mindfulness topics. In addition, new suggestions can additionally be generated by clicking the “new suggestions” button.

Additionally, the home screen features so-called “highlights of the day”, including blog articles with health-related topics, as well as promotion of events within hospital/institution [see Additional file 4E]. Moreover, the “open tasks” feature on the home screen summarizes all exercises and tasks that have been started once but never completed, pending assessments as well as all exercises and tasks marked as favorites [see Additional file 4E]. The “open tasks” feature functions as a reminder to continuously repeat the self-selected favorite exercises and tasks, to keep participants accountable and as such to promote the habit formation process. Lastly, participants have the opportunity to contact a trained coach specialized in physical activity, nutrition and mindfulness practices (via phone or email) from the Aeroscan company, in case of questions, technical issues, to inquire information on health-related topics or request support in forming habits [see Additional file 4F].

To further facilitate the habit formation process, two additional interactive worksheets (i.e., worksheet “cue selection” and worksheet “action planning”, [see Additional file 3C and D, respectively]) are sent to participants via newsletters (booking date + 14 and + 25, respectively). The worksheets feature information and prompt to a) determine everyday cues for action, which thus far did not result in automatic health behavior but in which participants wish to be more active, eat healthier or relax more in the future (e.g., during the lunch break, during medical rounds) and b) to develop if–then action plans for each of the three behaviors in order to specify when, where, and how an intended behavior will be executed (e.g., “If it is time for a lunch break, then I will eat an homemade salad”). The following criteria for choosing everyday situations (i.e., cues) have been formulated: something that a) occurs multiple times per week and b) with a given regularity.

#### Behavior change techniques

The behavior change techniques incorporated into the app align with the underlying theoretical model (Fig. 1) and can be subdivided in techniques targeting motivation (stage 1), volition (stage 2) and habit formation (stage 3). Table 1 gives an overview of the behavior change techniques covered by the app, the stages of behavior change, the mechanisms of action through which individual behavior change techniques have their effects [60] and how techniques are implemented in the app. All techniques are labelled according to the taxonomy of behavior change techniques (compiled by Michie and colleagues, [61]).

**Table 1:** Overview of behavior change techniques, mechanisms of action and implementation modes

Behavior Change Technique	Mechanism of action	Implementation mode
**Stage 1 – motivational**		
Goal setting (1.1) Goal setting outcome (1.3)	Behavioral regulation (intention) Goals	During the initial wizard, intended to tailor the app’s content at best possible to participants’ needs, work and private situation, participants are asked to set an individual health goal. An interactive goal setting worksheet is available to support participants in setting goals correctly. Note, participants are not specifically guided to differentiate between behavioral (e.g., set the goal of eating 5 pieces of fruit per day) and outcome goals (i.e., set a weight loss goals of 0.5 kg per week) but to choose a personally relevant health goal
Instruction on how to perform the behavior (4.1) Demonstration of the behavior (6.1)	Knowledge (self-efficacy) Beliefs about capability skills Social learning/imitating	The within-app exercise and task catalogue offers videos, written information and instructions on how to perform physical activity, and mindfulness behaviors. The videos feature a nurse executing the behavior correctly. By observing and imitating the nurse, self-efficacy is enhanced and beliefs are built that participants are able to achieve what the role model (i.e. nurse) achieved, raising expectations of success and motivating participants to work hard towards their goals
Information about health (5.1) and emotional (5.6) consequences	Knowledge Beliefs about consequences (positive, negative outcome expectancies) Attitude towards the behavior Perceived susceptibility/ vulnerability Intention Emotion	Blog articles (“highlights of the day”), covering different health topics related to physical activity, nutrition and mindfulness behaviors, are presented on the home screen and offering information about positive and negative health consequences, affecting participants’ knowledge, outcome expectancies, attitudes, perceived personal risk, emotional state and in turn their intentions. Additionally, “knowledge elements” of exercise and task suggestions included in within-app exercise catalogue offer information about health consequences that are supposed to have similar effects on aforementioned social-cognitive variables
**Stage 2—volitional**		
Action planning (1.4)	Behavioral regulation (action initiation)	Based on the goal setting instructions upon the initial wizard, participants are prompted via a newsletter sent to participants approximately 3 weeks post-baseline (booking date + 25), to make a plan by specifying how they want to achieve their intended behavior, what they want to do, where they want to do it and when. Action planning helps people to act in favorable situations and by anticipating contexts suitable for behavioral execution, which facilitates developing cue-behavior associations. Action planning as such can make cues more easily accessible in memory so that when exposed to the cue, it is more likely that the behavior is executed
Self-monitoring of behavior (2.3) Feedback on behavior (2.2) Discrepancy between current behavior and goal (1.6) Remove access to reward (7.4)	Behavioral regulation (action control) Knowledge	The home screen of the app features three progress bars (i.e., for physical activity, nutrition, mindfulness). Upon exercise/tasks completion a box can be ticked off. Ticking boxes (i.e., indicating completion of exercise/task) directly transfer to the progress bars, reflecting progress for the given behavior. Additionally, in case participants focus too much on one behavior and ignore another, negative progress is indicated in progress bars for the neglected behavior. Through this feedback and these gaming elements, participants can easily monitor their behaviors and goal progresses (positive, negative)
**Stage 3 – habit formation**		
Prompts/cues (7.1) Behavioral practice/rehearsal (8.1) Habit formation (8.3)	Memory, attention, decision processes Behavioral cueing Beliefs about capabilities Behavioral regulation	Via a newsletter participants receive an interactive worksheet stressing the importance of determining specific contextual cues (e.g., reoccurring situation or context such as lunch breaks) in which they intend to execute the behavior (e.g., stretching neck). Selecting triggers facilitates developing cue-behavior associations, which fosters the habit formation process. Additionally, the feature “open tasks” on the home screen continuously remembers participants to practice their favorite exercises and tasks
**Stage independent**		
Social support (unspecified) (3.1)	Social influences Social/professional role and identity	Participants have the possibility to contact a trained coach (via email or phone) in case of questions, concerns, problems. The coach could also function as a role model, providing information and knowledge on health-related topics or providing support for habit formation (e.g., provision of feedback)

*Note.* Numbers in parentheses for each behavioral change technique are per BCTTv1. *BCTTv1* BCT Taxonomy version 1 ([62])

### Evaluation design

**Fig. 3:**
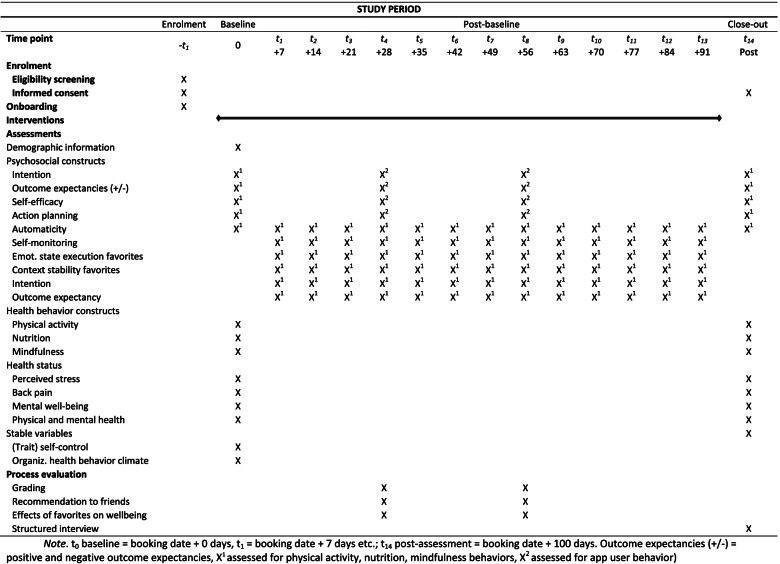
SPIRIT Schedule of enrolment, interventions, and assessments

A one-arm, multicenter mHealth interventional study will be performed. Participants complete all assessments in the app with the same assessment rhythm irrespective of when participants enroll in the study. Upon registration, participants can complete questions on socio-demographic variables, psycho-social determinants (e.g., intention, self-efficacy), physical activity, nutrition and mindfulness as well as health-related outcomes (e.g., back pain). Data will be collected across a 100-day period at four assessment points: baseline (booking date + 0), interim after 4 weeks (booking date + 28) and 8 weeks (booking date + 56) and post-intervention (booking date + 99). Additionally, 11 weekly assessments will be scheduled (booking date + 7, + 14, + 21, + 35, + 42, + 49, + 63, + 70, + 77, + 84, + 91). Participants will receive reminder emails for the baseline and post assessment four days after the respective assessment date (i.e., baseline reminder booking date + 4 days, post assessment booking date + 104 days). Reminders for the remaining assessments are incorporated into the app (via “open tasks”, see Description of the intervention for further information). Outcome data will be the same for all participants, whether they continue or discontinue/deviate from the intervention protocol. One week after the completion of the intervention, a structured interview is scheduled with volunteers to gain insights in participants’ experiences with the app (e.g., practicability, operability, acceptability). Due to economic reasons, the total set of items varies per assessment point. The Fig. 3 presents an overview of the enrolment schedule, the intervention, worksheets and assessments.

### Measurement instruments

Figure 3 presents an overview of the assessments points.

#### Demographic variables

In the baseline questionnaire participants’ age, sex, height, weight, education, profession and living with kids (if indicated with yes, the childcare situation is additionally assessed) are assessed. For age, height, and weight mean values and standard deviations as well as the body mass index (BMI) are calculated and frequencies are presented for the variables sex, education, profession and living with kids.

#### Psychosocial determinants of behavior change and habit formation

The following psychosocial determinants are assessed for physical activity, nutrition, and mindfulness behavior. Participants indicate their responses on a six-point Likert scale ranging from 1 (not at all true), 2 (not true), 3 (rather untrue), 4 (rather true), 5 (true) to 6 (absolutely true), if not otherwise specified. The baseline (t0), and post (t14) assessments focus on motivational (i.e., intention, outcome expectancies, self-efficacy) and volitional constructs (i.e., action planning), as well as on habit strength for physical activity, nutrition and mindfulness behaviors. The weekly assessments (t1, t2, t3, t4, t5, t6, t7, t8, t9, t10, t11, t12, t13) put focus on the habit formation process (i.e., self-monitoring, emotional states, context stability, intention, outcome expectancies) for all behavioral domains. Mean values and standard deviations are calculated to build the scales for the psychosocial determinants.

##### Intention

Intention is assessed with two items in accordance with Schwarzer, Schütz, Ziegelmann, Lippke, Luszczynska and Scholz ([63]; e.g., I intend to engage in at least 30 min physical activity with at least moderate intensity (i.e., mildly sweating) in the next four weeks on a nearly daily basis; I intend to eat at least five portions of fruit and vegetables daily in the next four weeks; I intend to practice mindfulness activities at least three times per week in the next four weeks, α = 0.56—0.79).

##### Positive and negative outcome expectancies

Outcome expectancies are assessed with six items (i.e., three positive and three negative) in accordance with Schwarzer, Schüz, Ziegelmann, Lippke, Luszcynska and Scholz [63]. Following the stem “If I engage in physical activity for at least 30 min with at least moderate intensity (i.e., mildly sweating)/ If I eat at least five portions of fruit and vegetables daily/ If I practice mindfulness activities for at least ten minutes daily… in the next four weeks…” participants indicate in how far they expect “to do something good for their health” (positive outcome expectancy) or “to have an additional obligation” (negative outcome expectancy; α = 0.52—0.72).

##### Self-efficacy

Self-efficacy is assessed with five items in accordance with Schwarzer and Renner [64]. Following the stem “I am sure I can engage in at least 30 min of physical activity with at least moderate intensity on an almost daily basis/I am sure I can eat at least five portions of fruit and vegetables/ I am sure I can practice mindfulness activities for ten minutes at least three times per week … in the next four weeks, even if,…” participants indicate how likely it is to engage in the respective behavior even if they are for example “tired” (α = 0.87—0.88).

##### Action planning

Action planning is assessed with two items in accordance with Schwarzer, Schüz, Ziegelmann, Lippke, Luszcynska and Scholz, [63]. Following the stem “For the next four weeks I have concretely planned, …” participants indicate for example “where”, “when” and “how often” they engage in physical activity, eat fruit and vegetables, and practice mindfulness activities (α = 0.79—0.83).

##### Automaticity

Automaticity is assessed with four items by means of the subscale Self-Report Behavioral Automaticity Index from the Self Report Habit Index (SRHI) [65, 66] at baseline and post assessments, and with two items at interim assessments (due to economic reasons). Following the stem “Physical activity/ Eating five portions fruit and vegetables/ Practicing mindfulness activities three times per week for ten minutes … is something” participants indicate on a five-point Likert scale ranging from 1 (do not agree) to 5 (totally agree) if they engage in the behaviors for example “automatically”. The SRHI has proven to be a reliable and valid instrument to measure habit strength for physical activity (α = 0.96) [66].

### Weekly assessments with regard to favorite exercises and tasks

#### Self-monitoring

Self-monitoring of self-chosen favorite exercises is assessed with one single-item for physical activity, nutrition, mindfulness and app-user behaviors. Following the stem “In the last 7 days, on how many days did you practice your favorite exercises?”, participants indicate the frequency of behavioral engagement according to the following scale: 0 (not at all), 1 (once), 2 (twice), 3 (three times), 4 (four times), 5 (five times), 6 (six times) and 7 (daily).

#### Emotional state while executing favorites

Emotional state is assessed with one single item (e.g., “How do you feel while practicing your favorite exercises) for physical activity, nutrition and mindfulness behaviors with response options in the form of smileys ranging from 1 (not at all good) to 5 (very good).

#### Context stability of favorites execution

Context stability is assessed in relation to the favorite exercises for physical activity, nutrition, and mindfulness behaviors with two items each (e.g., “I practice my favorite exercise always in the same context”) with response options ranging from 1 (not at all true) to 5 (absolutely true).

#### Intention

Intentions is assessed with one item (i.e., “I intend to practice my favorite exercise within the next 7 days”). Response options range from 1 (not at all true) to 5 (absolutely true).

#### Outcome expectancy

Outcome expectancy is assessed with one item (i.e., “How satisfied are you with the effects of your physical activity, nutrition, mindfulness favorites on your well-being?”). Response options range from 1 (not at all satisfied) to 5 (very satisfied).

### Health behavior

Changes in health behaviors is investigated from baseline to post assessments.

#### Physical activity

Physical activity is assessed with a shortened German version of the International Physical Activity Questionnaire (IPAQ, [67]) including four items, two for vigorous and two for moderate activities. As an example for vigorous intensity, following the stem “During the last seven days, on how many days did you do vigorous physical activities like heavy lifting, digging, aerobics or fast bicycling?” participants indicate vigorous physical activity frequency in days per week. Additionally, it is asked “How much time did you usually spend doing vigorous physical activities on one of those days? With participants indicating minutes of vigorous intensity per day. The IPAQ has proven to be a reliable and valid instrument in young and middle aged adults (36.8 ± 7.9 years; Spearman’s rank correlation coefficient clusters around 0.8, [68]).

#### Nutrition

Nutritional behavior is assessed through a Food Frequency List, adapted and proven to be valid, according to Winkler and Döring [69].The food frequency list entails 27 food items (e.g., fish, cake, fast-food) for which participants are asked to indicate on a six-point Likert scale ranging from 1 (almost daily), 2 (multiple times per week), 3 (once per week), 4 (multiple times per month), 5 (once per month) to 6 (never) how frequently they consume each item respectively. Spearman’s rank correlation coefficients range between 0.15 (sweets and candies) and 0.60 (curds, yoghurt, sour milk, milk, butter milk, mineral water.

#### Mindfulness

Mindfulness is assessed with a short and psychometrically sound version of the Freiburger Mindfulness Inventory [70, 71]. Participants are asked to rate each of the 14 items (e.g.,”I am open to the experience of the present moment”) while taking into account the last four weeks (α = 0.89). Responses are given on four-point-Likert scale ranging from 1 (rarely), 2 (occasionally), 3 (fairly often) to 4 (almost always). The mean value of all items is calculated to build the scale.

### Health status

Changes in health status are examined from baseline to post assessments and mean values will be calculated to build each scale.

#### Perceived stress

Perceived stress is assessed with the Perceived Stress Scale (PSS-4 [72],), a short instrument assessing the degree to which situations in a person’s life over the past month are appraised as stressful. Participants are asked to rate their feelings and thoughts (e.g., “In the last month, how often have you felt that you were unable to control the important things in your life?”) during the last months (α = 0.80—86). Responses are given on a five-point-scale range from 0 (never) to 4 (very often). The PSS-4 has been shown acceptable psychometric properties [73].

#### Back pain

Back pain is assessed with two single items. Participants are asked to rate their back health with response options ranging from 1 (bad), 2 (not good), 3 (satisfactory), 4 (good) to 5 (very good) and to indicate if they suffered from back pain within the last three months (response options: yes – no).

#### Mental well-being

Mental well-being is assessed with the World Health Organization—Five Well-Being Index (WHO-5), a short self-reported measure of current mental wellbeing. For the German version, excellent psychometric properties have been reported [74]. The WHO-5 includes five statements, which participants rate in relation to the past two weeks and according to the following scale: 0 (at no time), 1 (some of the time), 2 (less than haft of the time), 3 (more than half of the time), 4 (most of the time) and 5 (all of the time). The total raw score, ranging from 0 to 25, is multiplied by the factor four to give the final score, with 0 representing the worst imaginable well-being and 100 representing best imaginable well-being. Internal consistency has been shown to be very good (Cronbachs` α = 0.92).

#### Physical and mental health status

Physical and mental health status are each assessed with one single item. Participants are asked to rate their general physical and their general mental health status with response options ranging from 1 (very bad), 2 (bad), 3 (moderate), 4 (good) to 5 (very good).

### Stable variables

Stable variables will be assessed at baseline only. Mean values will be calculated.

#### Self-control

Trait self-control is assessed at baseline by means of the brief, German version of the Self-Control Scale (SCS-K-D [75, 76],) containing 13 items (e.g., “I am good at resisting temptation”), which participants rate according to the following scale: 1 (not at all true) to 5 (totally true). The brief version has shown to be reliable and valid ([75]; α =  ≥ 0.93).

#### Organizational health climate

Exercise, eating and mindfulness climate are assessed by the validated Organizational Health Behavior Climate scale (OHBC, [77]) using the organizational practices subscale only (i.e., 4 items per behavioral domain). Given that the OHBC features exercise and nutrition behaviors only, respective items for mindfulness behaviors were added. Participants rate their organizational exercise, nutrition and mindfulness practices (e.g., “In this organization, there are posters featuring exercise and physical activity/brochures and information on the Internet about healthy nutrition/posters with mindfulness practices”) according to the following scale 1 (I do not agree at all) to 5 (I fully agree). Internal consistency has been reported for exercise and eating behaviors, respectively (α = 0.82 – 0.89).

### Process evaluation

Participants satisfaction and experiences with the app will be evaluated using a mixed-methods approach (i.e., questionnaire and structured interview). Questionnaire items assess the whole set of psychosocial items (i.e., motivational, volitional and habit formation process-related items with regard to app use) at interim assessments at week four and eight (booking date + 28 and + 56, respectively), only, and not at baseline, given that participants are not familiar with the app at study start (t0). Items derive from the respective instruments described above and participants indicate their responses on a six-point Likert scale ranging from 1 (not at all true), 2 (not true), 3 (rather untrue), 4 (rather true), 5 (true) to 6 (absolutely true), if not otherwise specified. Mean values are calculated for each scale.

#### Intention

Intention is assessed with two items (e.g., I intend to use the app at least daily in the next four weeks).

#### Positive and negative outcome expectancies

Outcome expectancies are assessed with six items (i.e., three positive and three negative). Following the stem “If I use the app at least once daily in the next four weeks…” participants indicate in how far they expect “to do something good for their health” (positive outcome expectancy) or “to have an additional obligation” (negative outcome expectancy).

#### Self-efficacy

Self-efficacy is assessed with five items. Following the stem “I am sure I can use the app at least once daily in the next four weeks, even if, …” participants indicate how likely it is to use the app even they are for example “tired”.

#### Action planning

Action planning is assessed with two items. Following the stem “For the next four weeks I have concretely planned, …” participants indicate for example “where”, “when” and “how often” they use the app.

#### Automaticity

Automaticity is assessed with two items by mans of the Self Report Habit Index. Following the stem “Using the app once daily is something …” participants indicate on a five-point Likert scale ranging from 1 (do not agree) to 5 (totally agree) if they engage in the behaviors for example “automatically”.

#### Grading

Participants are asked to rate the app using the following scale (equivalent to the German grading system, “How would you grade the app”): 1 (very good), 2 (good), 3 (satisfactory), 4 (sufficient), 5 (inadequate) to 6 (insufficient).

#### Recommendation to friends

Participants are asked to indicate on a single item whether they would recommend the app to friends and colleagues (e.g., “Would you recommend the app to friends, acquaintances and colleagues that work in the same field) with responses ranging from 1 (no), 2 (rather no), 3 (neither…nor), 4 (rather yes) to 5 (yes).

#### Semi-structured interview

Upon completion of the post-assessment (100-days post baseline) participants are invited to voluntarily take part in an interview (approximate duration 30–60 min). Main topics of the interview are: experiences with app in general, experiences with operability of app (e.g., setup), experiences with practicability (e.g., in the work context), app user behavior, opinion about personal relevance and effects of the app on health behavior. Reasons for dropout will be evaluated for participants who withdrew during the intervention period. An overview of the main interview questions is presented in Table 2.

**Table 2:** Overview of semi-structured interview questions about participants’ experiences with the Habit Coach app (process evaluation)

Topic	Question
General experiences with app	What are your experiences with the app? Please elaborate.
Experiences with operability of app (e.g., setup)	How do you perceive the operability (e.g., setup of menu) and features of the app?
Experiences with practicability (e.g., work context)	How practicable do you perceive the app during your working routine?
App user behavior	How, how often, when and where do you preferably use the app?
Opinion about personal relevance	As how personally important and relevant do you perceive the app?
Effects of app on health behavior	What effect does the app have on your health behavior? Have you made any changes in your behaviors due to using the app? Please also think about any spontaneous negative events or unintended effects of the app. Please elaborate.

### Data management

All data are assessed through the mobile app, which will be extracted upon intervention completion in Excel files, before they are transferred in SPSS. All data collected during the various assessment waves will be stored in separate SPSS files, one file per assessment point. After data collection completion, data will be transformed in one SPSS file based on the participants’ identification numbers. Variable labels will be defined in SPSS. Additionally, information about the variables assessed is gathered in an excel codebook including variable names, labels, codes, specific item information etc. To prevent drop out, user will be contacted through reminder emails. Moreover, incomplete assessments and tasks will continuously be displayed on the home screen (i.e., “open tasks” feature). To ensure that instruments used are reliable and valid, only validated questionnaires will be utilized. To overcome data loss, all data will be stored on the servers of the Aeroscan company, which are hosted by the Domain Factory in Germany (domainfactory GmbH, Oskar-Messter-Str. 33, 85,737 Ismaning, Germany). The Domain Factory ensures reliability and unauthorized access to the servers. All data will be separately and confidentially stored at two different data bank systems and in line with the German data protection ordinance, ensuring a strict separation between the user’s identity (i.e., contact data) and psychosocial and demographic data. Only a few specifically trained Aeroscan IT experts can access the data. The few people with data access signed a confidentiality agreement. As long as the data are not effectively anonymized, no data usage will be conducted by the Aeroscan company, the company health care insurance or else. Only the research team (i.e., two professors, one post-doctoral researcher, one student assistant) has access to the data until anonymization. Upon termination of the study, all user data will be deleted.

Participants will be informed on every aspect of the study that concerns their participation (e.g., data collection, storage, anonymization, access to data, participant rights etc.) and have to give written informed consent to enter the program (i.e., without informed consent, access to the app is denied). With respect to data sharing activities and distribution of study results, participants are informed that data are always shared on group level means and never as individual results (e.g., in (inter-)national peer-reviewed publication, conference abstracts) and that interview data will always be pseudonymized. Lastly, the data collection will be monitored and guided by the Aeroscan company in close collaboration with the researchers. Given that this study does not involve patients, no data trial steering or data monitoring committee was assigned.

For the semi- structured interviews a coding list will be used that links participants’ names with respective interview data. Only the research team has access to this list, which will be stored, separately from the interview data, in a looked cupboard in the university. Upon interview transcription, the coding list will be deleted and the interviews will be anonymized.

### Statistical power

The required sample size for the primary outcome (automaticity over time) was estimated based on recently published recommendations [78]. The power estimation revealed that a level-2-sample size with at least *N* = 228 participants (i.e., number of required individuals, accounting for 30% sample attrition) and a level-1-sample size of at least *n* = 13 measurement points (i.e., number of repeated measurements) is required to detect medium-sized population effects of *f* = 0.40 [21, 79] with sufficient power (≥ 0.80).

### Statistical analysis

Statistical analysis will be performed using SPSS Version 27 and conducted after completion of the data collection process. Interim analyses will not be performed. All primary outcome data will be screened for normal distribution by using the Shapiro–Wilk test. Data will be checked for outliers and missing data. Descriptive statistics will be run to explore the sample characteristics. Continuous variables will be presented as means and standard deviations, while categorical variables will be displayed by percentages of participants in each possible category. Intention-to-treat analyses will be conducted, given that drop-out rates are usually high in eHealth research [80], per protocol analysis are not feasible. Only data sets from participants who completed the baseline, post and 50% of the weekly assessments will be included in the analysis. To investigate difference between the sample of analysis and those who provided baseline data only, differences between baseline scores and a dichotomous attrition variable (i.e., 0 = not retained for analysis, 1 = retained) will be analyzed using χ^*2*^ and *t*-tests, followed by logistic regressions. To test for changes in habit strength, motivational (intention, self-efficacy, and outcome expectations), volitional constructs (action planning), health behaviors and health status from baseline to post assessments, analysis of variance with repeated measures and *t*-tests between subsequent measures will be run (hypothesis 1 a-e). To examine hypotheses 2–4, hierarchical multiple linear regression analyses will be conducted, with motivational, volitional and habit-related predictors at baseline to predict habit strength, health status and health behavior at post-assessment. To account for the hierarchically structure of data (i.e., repeated measurements nested within individuals) we plan to run two-level models with 13 assessment periods nested in participants (hypotheses 5 a-b, linear mixed models, LMM, [81]). LMM allows to test for simultaneous assessment of the effects of within-person variation in weekly assessments of behavior,habit strength and predictor variables (level 1) and between-person variables such as age, health status and other covaraites measured only once (level 2). Prior to running analyzes, the time parameters will be grand mean centered to minimize multicollinearity. Subsequently, various steps will be undertaken to determine appropriate model fit, as suggested by Field [81]. Once the model shows appropriate fit parameters, LMM can be conducted by selecting the Restricted Maximum Likelihood for estimation method [81]. In a model including all behavior-related cognitions and covariates simultaneously, motivational and volitional variables will be modeled at the between-person level (e.g., comparing persons with higher average versus lower average intentions) and the within-person level (e.g., assessments when intentions to engage in health behavior were higher-than-usual vs. lower-than-usual). Models will be run separately for each behavioral domain.

### Adverse effects

Adverse effects are defined as negative outcomes associated with participation in the current trial. Possible adverse effects due to study participation might be injury resulting from increased physical activity. The occurrence of any adverse effects will be tracked and evaluated via the semi-structured interview.

### Dissemination

The results of this trial will be published in international peer-reviewed journals and presented on national and international conferences. Additionally, the trial has been pre-registered and is publicly available by the German Clinical Trials Register and the World Health Organization. All relevant data (in accordance with the informed consent form) from this study will be made available upon study completion.

## Discussion

### Overview and implications

Irregular and long work schedules often prevent health care professionals from regular engagement in health promoting behaviors [7]. Promoting health enhancing behaviors in health care professionals is therefore of upmost importance. This trial addressed this call by taking into account the adverse working conditions of health care professionals, as well as acknowledging that behavior change is not solely a product of deliberative psychological but also of non-conscious processes. Through a mHealth intervention fostering healthy habit formation for physical activity, nutrition and mindfulness practices, and easily integrable theory- and evidence-based strategies facilitative of habit formation, shortcomings of previous research are overcome. We examine whether the mHealth intervention can increase participants’ habit strength for physical activity, nutrition and mindfulness behaviors. In addition, it will be examined whether potential changes in habit strength are predicted by changes in psychosocial variables. Through the weekly assessments we are able to seek insights into participants’ weekly struggles to adopt a healthy way of living.

### Strengths, challenges and limitations

Strengths. The mHealth intervention is theory driven and provides support during the entire behavior change process, starting with a motivational (i.e., goal setting), over a volitional (i.e., action planning) to habit formation and behavioral maintenance phase. Although mHealth interventions intended to increase physical activity and eating behaviors have shown positive effects, these effects often times were small and highly variable, caused by insufficient description of theoretical foundations and behavior change techniques used. The present study focuses specifically on mechanisms of action and respective behavior change techniques in order to predict change in behavior and make intervention design and evaluation more transparent and replicable. Furthermore, by allowing health care professionals to integrate the within-app mini interventions into their daily (work) routine, it is intended to tackle commonly reported problems of implementing programs such as massive disruption of the working day and attrition based on problems to fit interventions into daily routines outside the work context.

Limitations. The study design is not without limitations. First, we are not able to collect participants for a control group, as the resources for this study are limited. Consequently, it is not possible to evaluate the effectiveness of the mHealth app. Furthermore, due to the uncontrolled design it is not possible to help close the knowledge gap pertaining to the question, as to whether traditional context-dependent repetition is necessary for habit formation or if it represents the most promising or reliable ingredient of habit formation interventions. Future studies are warranted to compare and evaluate habit formation interventions against non-habit based programs. Second, the mHealth evaluation is also limited given that no follow-up period is included. Whether habits indeed last over time and so facilitate behavioral adherence, cannot be confirmed without (or even with short) follow up periods [29].Third, in order to test our hypotheses participants will need to take part in many assessments, which might result in high attrition rates. We try to prevent dropouts by sending email reminders and keeping incomplete questionnaires available on the home screen under “open tasks”. Lastly, as the researcher(s) who analyze the data will also be involved in the data-collection process and in the study design, blinding of the analyst is not feasible. In the next step, the summative evaluation of the Habit coach app against a control group in a randomized controlled trial is warranted.

In sum, this trial addresses health care professionals’ adverse working and health conditions and acknowledges that health behavior change is a results of deliberative as well as non-deliberative psychological processes. Through a mobile app fostering healthy habit formation in the areas of physical activity, nutrition and mindfulness behaviors and theory-based strategies to support habit formation, shortcomings of previous research are overcome. The analyses and evaluation of the app will provide insights in the habit formation process supported by the app and will contribute to the mHealth literature in both theoretical and practical aspects.

## Supplementary Information

**Additional file 1.** SPIRIT 2013 Checklist: Recommended items to address in a clinical trial protocol and related documents. SPIRIT Checklist Items.

**Additional file 2.** The TIDieR (Template for Intervention Description and Replication) Checklist. TIDieR Checklist Items.

**Additional file 3.** Interactive Worksheets. Worksheets provided by researchers.

**Additional file 4.** Screenshots. Screenshots of the App.

## Data Availability

Not applicable.
