# Supplementary material for: Study protocol for the description and evaluation of the “Habit Coach” - a longitudinal multicenter mHealth intervention for healthy habit formation in health care professionals
Source: BMC Public Health. 2022 Sep 4;22:1672. doi: 10.1186/s12889-022-13986-0 (PMC9440859; doi:10.1186/s12889-022-13986-0)
Supplement: Supplementary file 3 — Additional file 3. Interactive Worksheets. Worksheets provided by researchers. [file 12889_2022_13986_MOESM3_ESM.pdf]

Additional File 3. Interactive Worksheets

Additional File 3 A. Worksheet “Selecting favorites”.

Additional File 3 B. Worksheet “Goal Setting”.

Additional File 3 C. Worksheet “Cue Selection”.

Additional File 3 D. Worksheet “Action planning”.

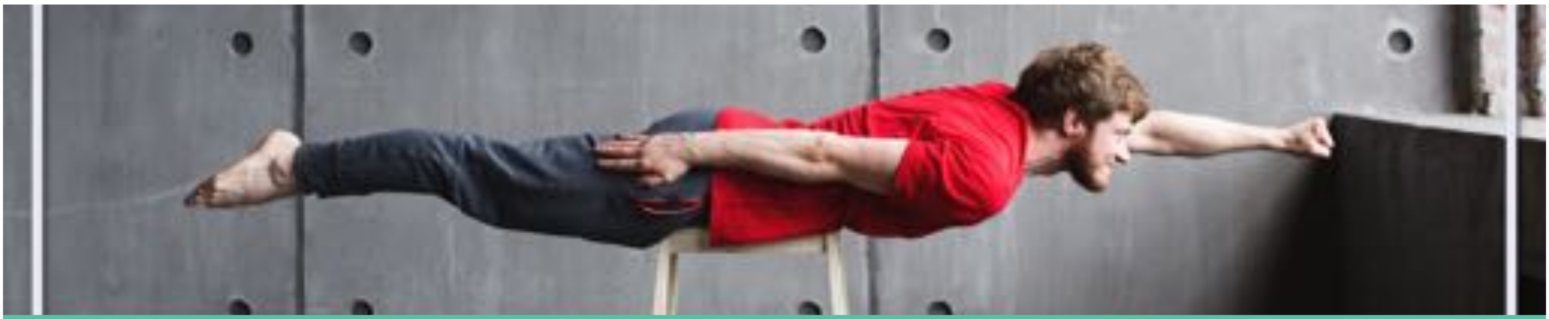

SELECT YOUR FAVORITE EXERCISES FOR NEW ENERGY IN EVERYDAY LIFE

How favoring exercises can help you form habits.

To be able to form habits and better achieve a desired health behavior it is advisable, after a period of trial an error, to mark your favorite exercises from the app as favorites and repeat these exercises regularly instead of constantly doing new ones. By repeating the same favorite exercises, it is more likely that these exercises will become a habit.

We recommend that you set your favorites in the first four weeks of app use and repeat only those favorites from that point on.

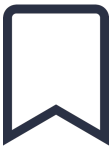

You can easily set favorites by clicking in this icon. Think carefully about which exercises you liked, as these favorites will stay with you for a while and ideally remain part of your everyday life even after you've finished using the app.

When choosing favorites, it is helpful to pick favorites from each area (nutrition, mindfulness and physical activity).

Do you already have an idea which exercises you would like to choose as a favorite? Feel free to use the space to make your own notes on the favorites.

____

____

____

____

____

____

____

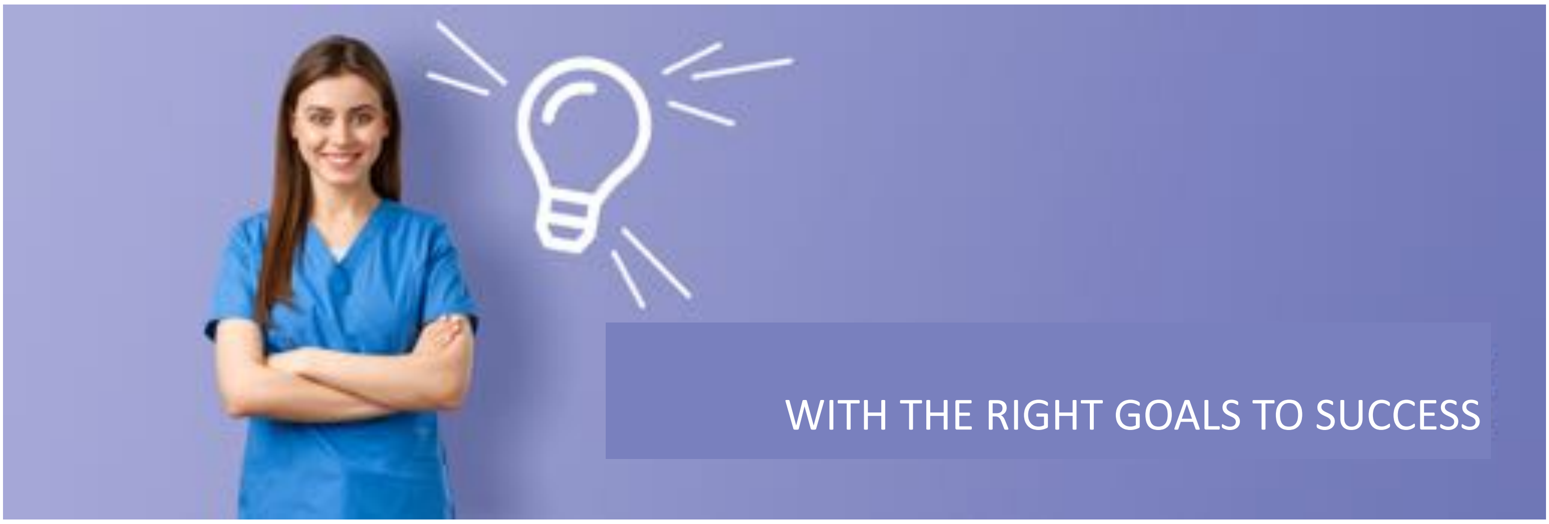

WITH THE RIGHT GOALS TO SUCCESS

Would you like to be more active, be more relaxed or change something about your diet? We can help you achieve your goals in all areas.

In order to make success tangible and to be able to stay on track, it is helpful to have concrete and realistic goals. They help you stay on track in the long run and keep you motivated. Read here how to set realistic goals using the SMART formula and enter your personal goals below.

S - specific: The more precisely you formulate your goal, the better you will succeed in realizing it.

> *Ex.: I would like to be physically active in my free time for at least 2 hours every week.*

M - measurable: Your goals should be set in such a way that you can easily measure them. Measurable goals allow you to evaluate whether you have achieved your goals or not.

> *Ex.: Walk 5 kilometers briskly.*

A - achievable: Goals do not always have to be set high, but should be realistic in the first place.

> *Ex.: Currently I manage to cook something healthy once a week. Next week, I will try to cook something healthy twice a week.*

R - relevant: The goals should be personally meaningful to you so that you are willing to stick to your goal.

> *Ex.: After a tiring shift, I take 20 minutes for myself and read in my book.*

T - time-bound: If you determine exactly by when you want to achieve your goal, you can work towards it and stay on track.

> *Ex.: In one month, I would like to take home-made food to work instead of eating in the canteen*

Now that you've read about ideal goal setting, it's your turn to set your own goals in the areas of nutrition, physical activity and mindfulness for the coming weeks. Think of the SMART formula and formulate a goal for each of the three areas. You will need those goals later.

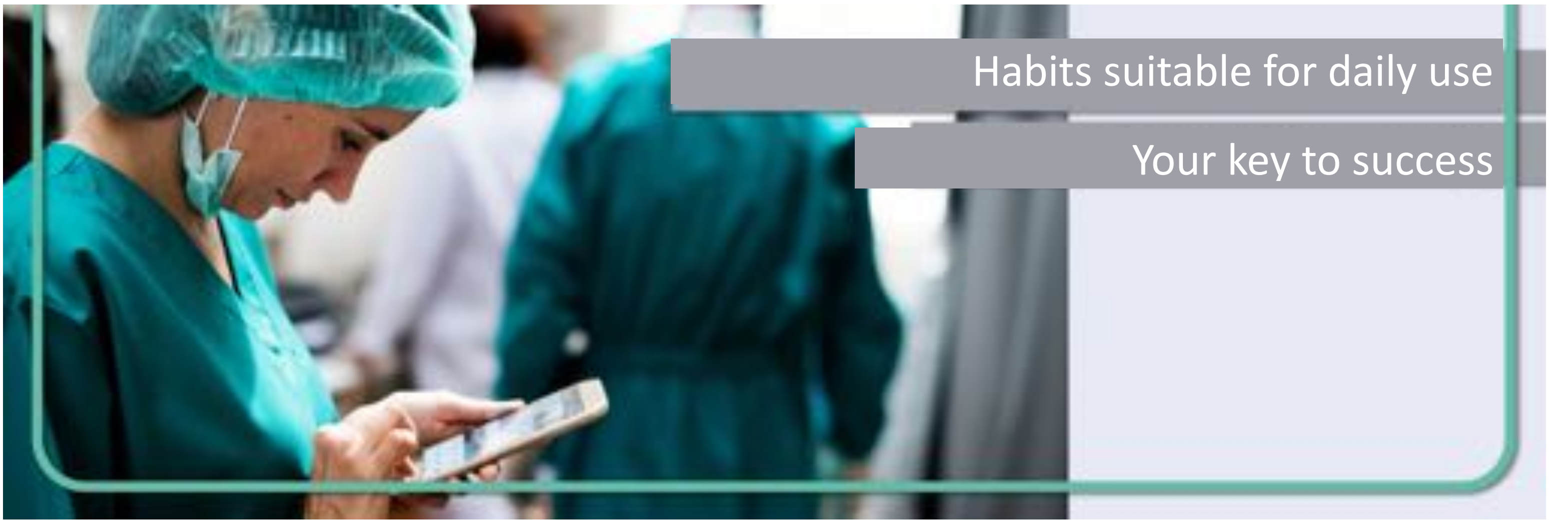

What?
Habits are actions that you do over and over again without having to think explicitly about how they need to be executed. For example, brushing your teeth in the morning, tying your shoes or reaching into the cookie jar during shift handovers. When choosing habits, one should always make sure that they are conducive to good health. This way, they can help you have more energy during stressful times.

Why?
Habit formation can save cognitive resources, which then can be used for other tasks that require more attention. For example, when riding a bicycle, you can focus your attention completely on the traffic because riding a bicycle has already become a habit.
Furthermore, certain behaviors can be performed more quickly through habit formation because there is no need to create new action plans for the behavior.

How?
To form a habit, it usually takes 66 days. Only then can a regular action become a habit. In the app, setting favorites helps you focus on a smaller number of exercises that are relevant to you and that can be performed regularly over the course of the program and thus become a habit.
The key here is that the action that is to become a habit is preferably always associated with the same trigger. Triggers can be chosen flexibly, but should occur as frequently as possible in your everyday life so that habit formation can take place continuously. Examples of triggers can be: sorting medicine or the nightly round.

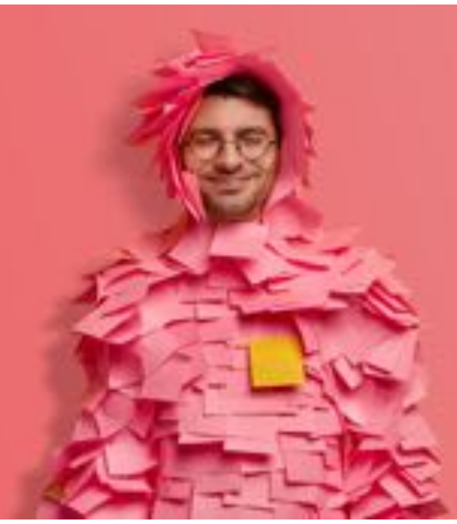

WITHOUT A PLAN?
The path to greater well-being does not always have to be complicated.

How can you develop specific plans for yourself?

In order to achieve your goals with the help of habits, it is advisable to set up concrete plans to reach your goals. Plans in if-then formats are particularly useful for planning and are structured as follows: The if-part describes the trigger, i.e. a situation in your everyday life that occurs regularly (e.g.sorting medicine), that triggers a desired action (e.g. perform favorite exercise #1). The then-part represents the action itself that is to be triggered by the trigger. Here`s an example: If I am on the search for a snack, then I will eat a banana first.
In the beginning, you will still consciously think of your if-then plans. Over time, these will become automated and the actions will become habtiual.

To plan your individual health goals, think about the favorite exercises you have chosen and construct if-then plans to match them.<br>You can also include the triggers you selected last week.<br><br>An example of the favorite “Fit back: Neck stretch“ would be:<br>„If I am sitting in shift handovers, then I will stretch my neck for 2 minutes“

Physical activity

Favorite:	
If:	Then:
Favorite:	
If:	Then:
Favorite:	

If:	Then:

Nutrition

Favorite:	
If:	Then:
Favorite:	
If:	Then:
Favorite:	
If:	Then:

Mindfulness

Favorite:	
If:	Then:
Favorit:	
If:	Then:
Favorit:	
If:	Then:
