# Supplementary material for: Study protocol for the description and evaluation of the “Habit Coach” - a longitudinal multicenter mHealth intervention for healthy habit formation in health care professionals
Source: BMC Public Health. 2022 Sep 4;22:1672. doi: 10.1186/s12889-022-13986-0 (PMC9440859; doi:10.1186/s12889-022-13986-0)
Supplement: Supplementary file 4 — Additional file 4. Screenshots. Screenshots of the App. [file 12889_2022_13986_MOESM4_ESM.docx]

**Additional File 4. Screenshots of the features of the Habit Coach app**


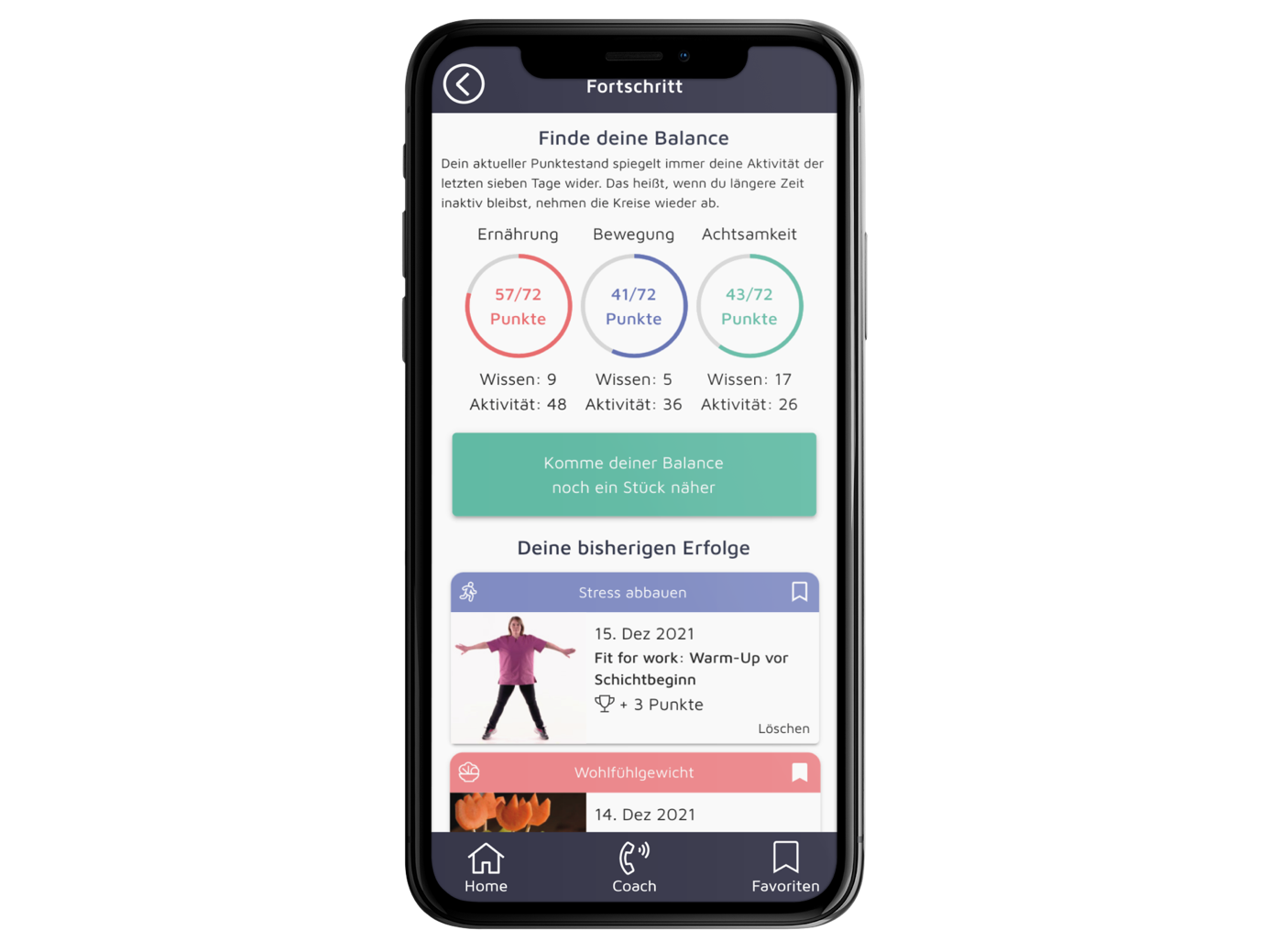


**[3]** Completed tasks

**[2]** Option to generate new exercise and task suggestions to achieve progress in participants’ balance. Engaging in suggested tasks will positively impact the respective progress bar - Discrepancy between current behavior and goal (1.6) & Remove access to the reward (7.4)

**[4]** Option to mark exercise or task as a favorite

**[1]** App theme “well-being through balance” displayed as progress bars for each behavioral domain, including details on achieved points - Feedback on behavior (2.2) & self-monitoring of behavior (2.3)

[4]

[3]

[2]

[1]

**Additional File 4A. Progress bar and bookmark symbol.** Numbers in parentheses for each behavioral change technique are per BCTTv1. *BCTTv1* BCT Taxonomy version 1 (Michie et. al., 2013).


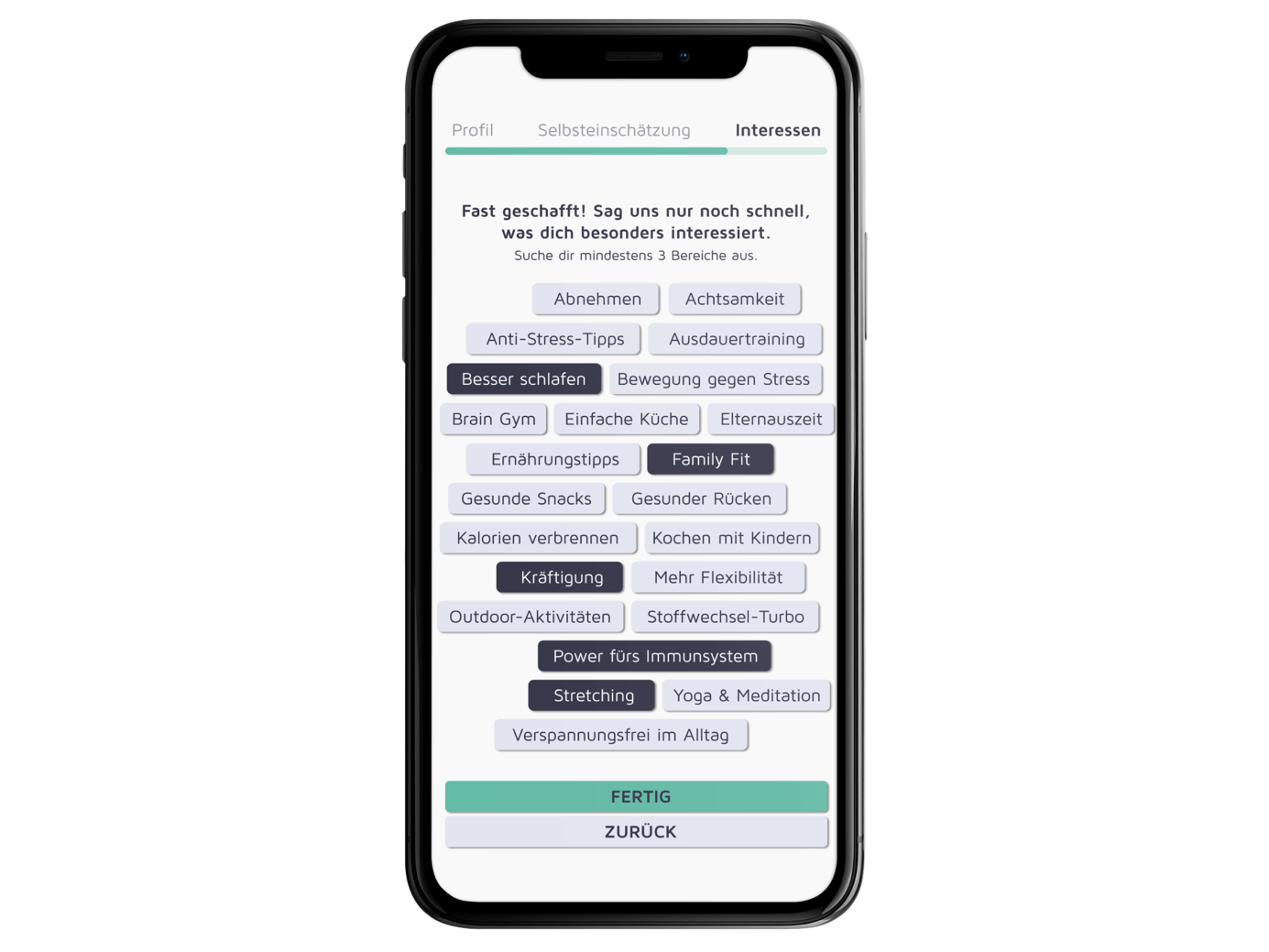

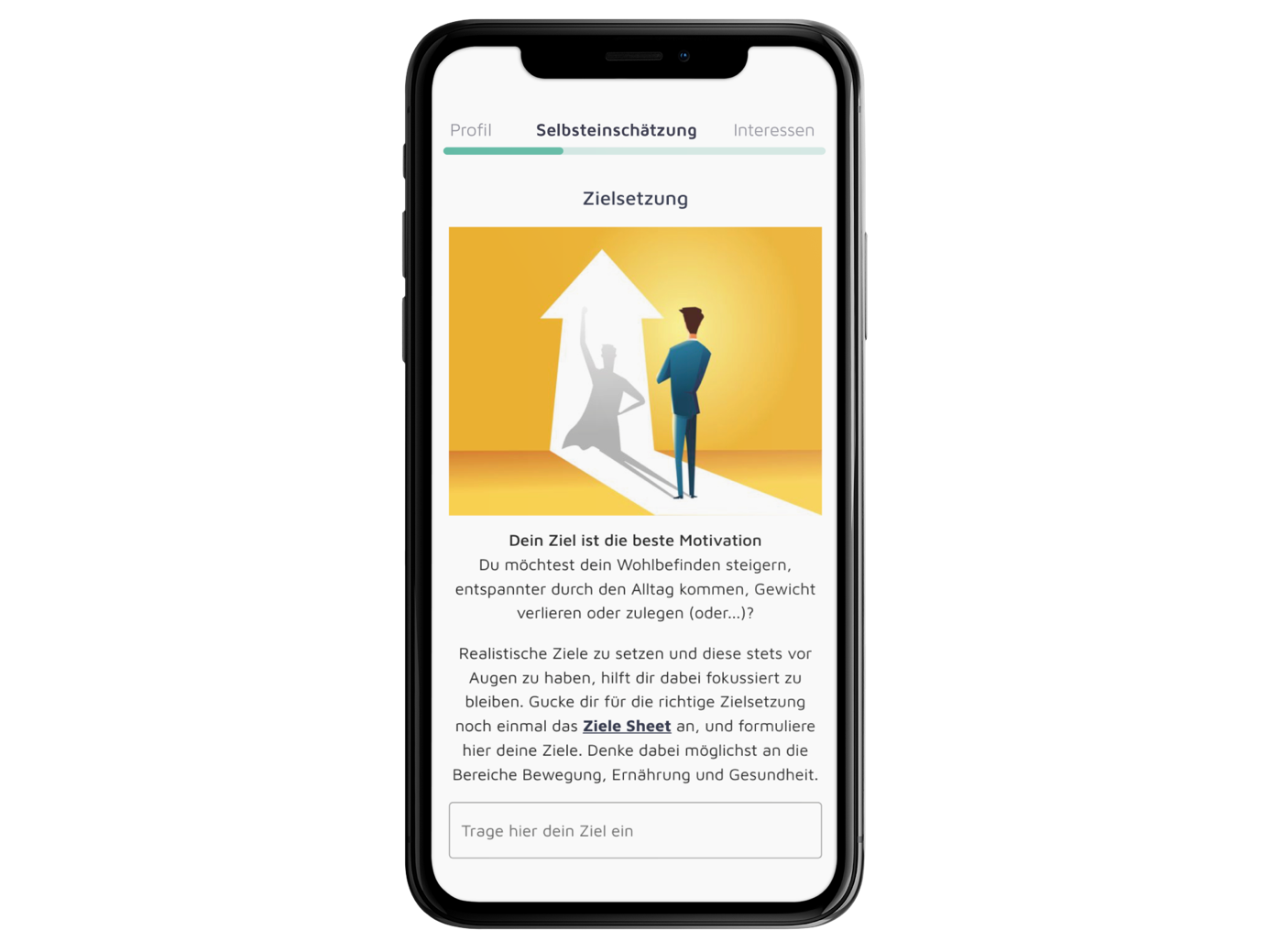


**[5**] Wizard to tailor app content to participants needs and interests

**[6]** Instruction on how to set goals including a link to the goal setting worksheet - goal setting (1.1)

**[7]** Possibility to enter individual health goal - goal setting (outcome) (1.3)

[5]

[7]

[6]

**Additional File 4B. Wizard and setting individual health goal.** Numbers in parentheses for each behavioral change technique are per BCTTv1. *BCTTv1* BCT Taxonomy version 1 (Michie et. al., 2013).


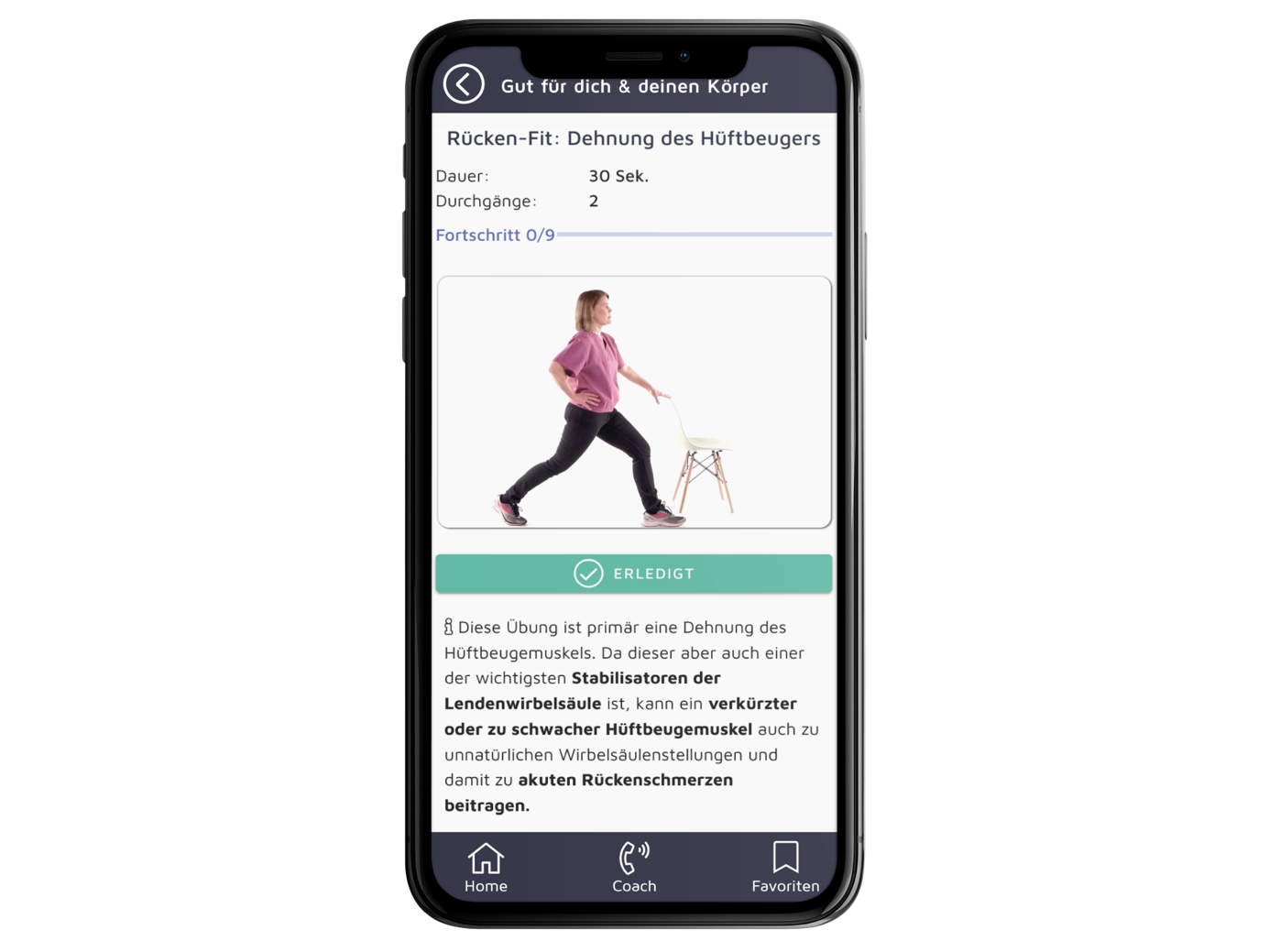


[8]

**[10]** Written explanation on how to perform the behavior - Instruction on how to perform the behavior (4.1)

[10]

[9]

**[9]** Videos or pictures demonstrating the behavior - Demonstration of the behavior (6.1)

**[8]** Name of the exercise or task

**Additional File 4C. Within-app exercise catalogue and example of exercise and task suggestions.** Numbers in parentheses for each behavioral change technique are per BCTTv1. *BCTTv1* BCT Taxonomy version 1 (Michie et. al., 2013).


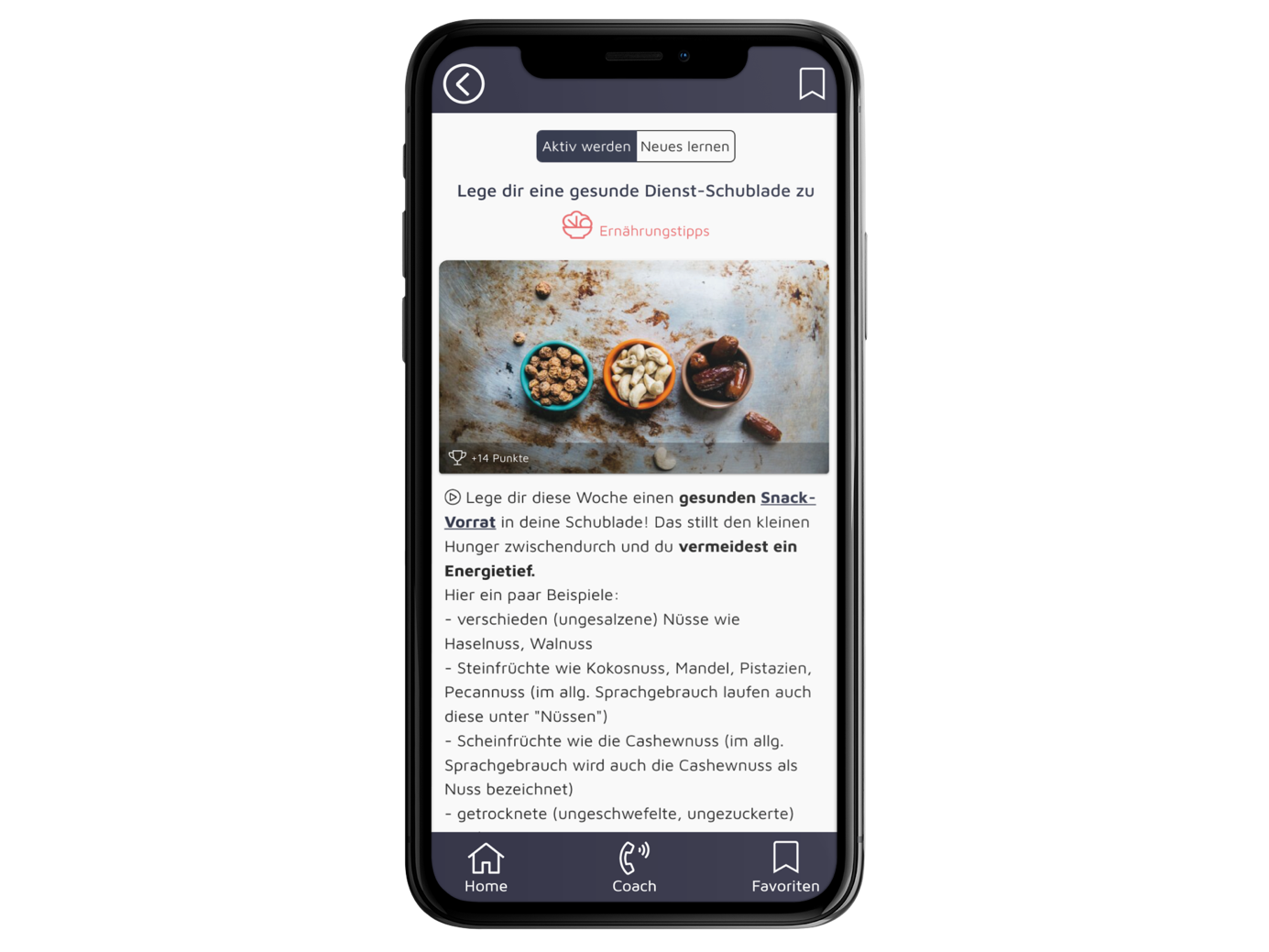

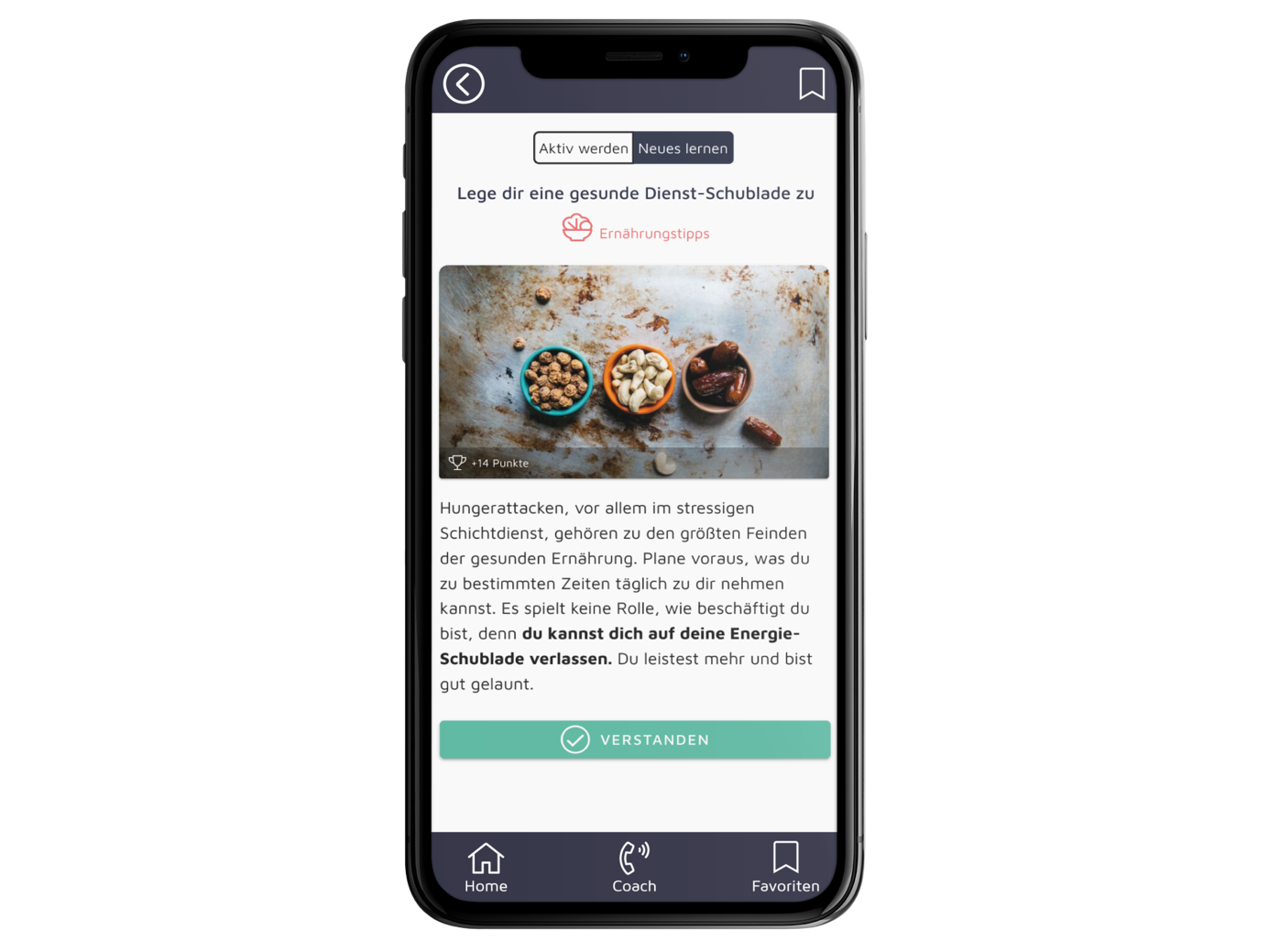


**[12]** Knowledge element to motivate participants to learn something new - information on health and emotional consequences of a recommended behavior (5.1 & 5.6)

**[13]** Button to mark task as completed

**[11]** Activity element to motivate participants to take action - instruction on how to perform the behavior (4.1)

[13]

[12]

[11]

**Additional File 4D. Exercise and task suggestions consisting of activity and knowledge elements.** Numbers in parentheses for each behavioral change technique are per BCTTv1. *BCTTv1* BCT Taxonomy version 1 (Michie et. al., 2013).


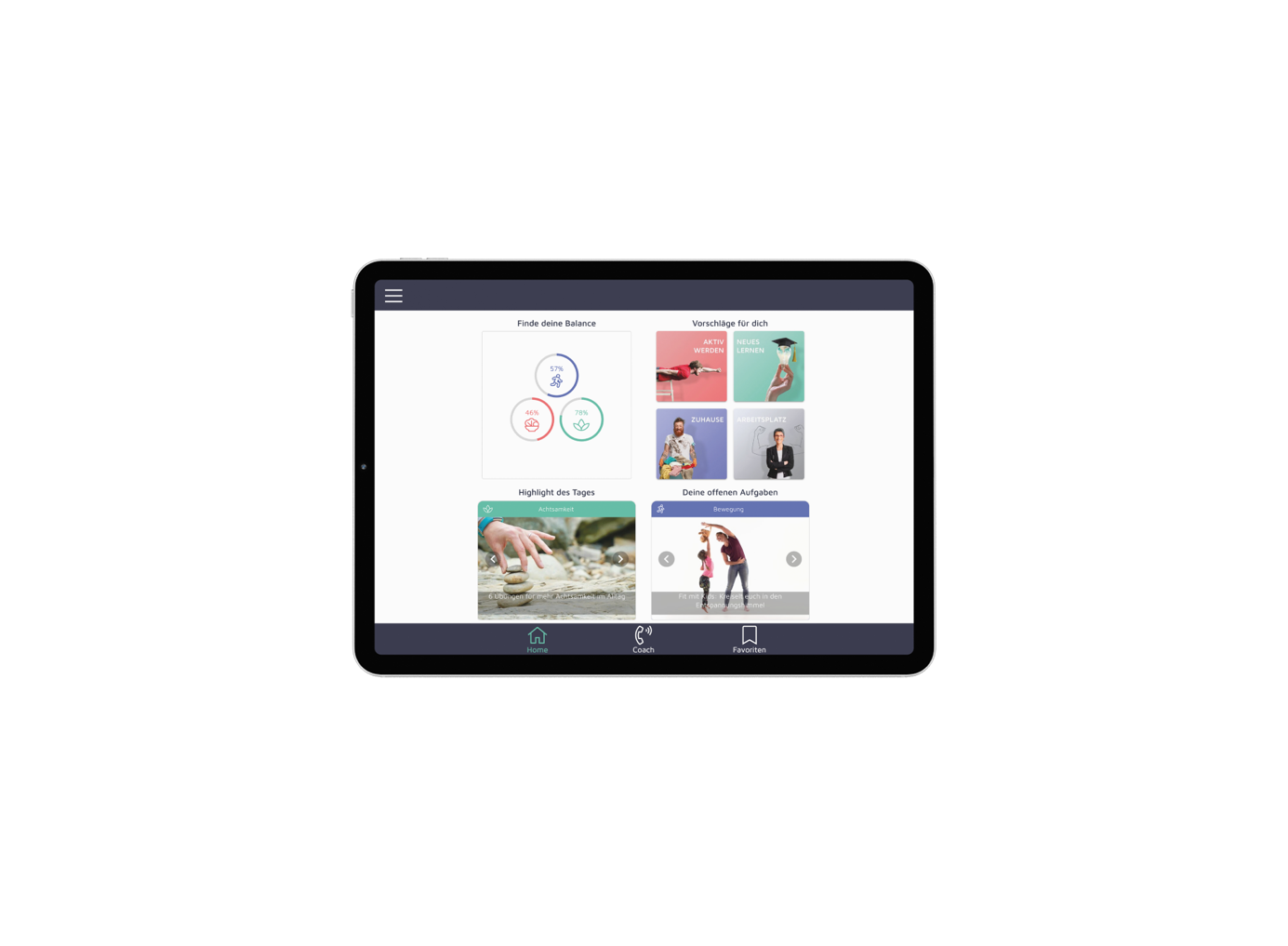


[14]

[17]

[16]

**[14]** Progress bars

**[16]** “Highlight of the day” including blog articles with health-related topics and promotion of events within institution

**[15]** Exercise and task suggestions

**[17]** “Open tasks” display incomplete tasks, reminders - prompts/cues (7.1)

[15]

**Additional File 4E. Home screen featuring highlights of the day, exercise and task suggestions and open tasks.** Numbers in parentheses for each behavioral change technique are per BCTTv1. *BCTTv1* BCT Taxonomy version 1 (Michie et. al., 2013).


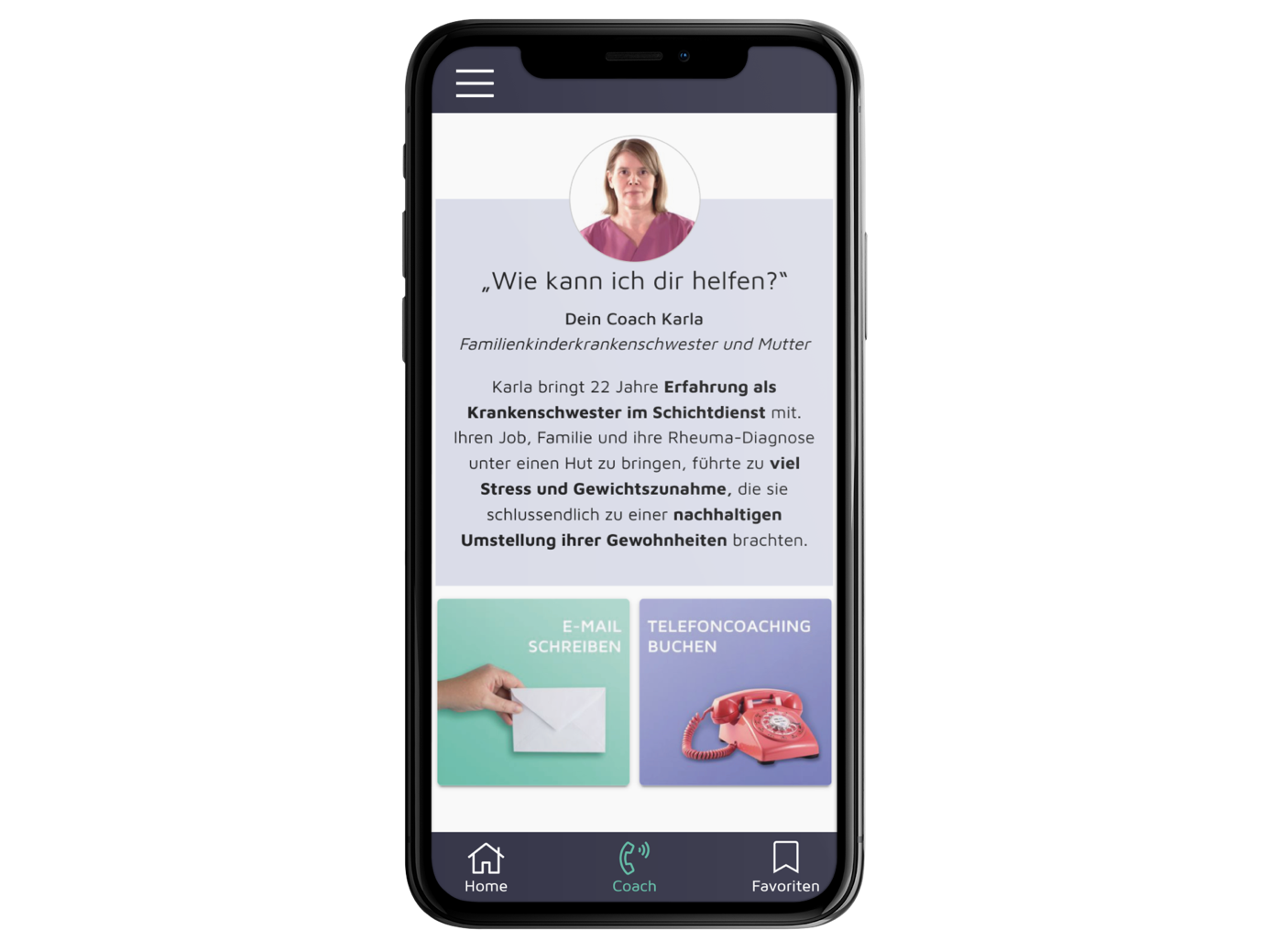


**[18]** “How can I help you?” Optional choice for contacting a coach via e-mail or telephone - social support (unspecified)

(3.1)

[18]

**Additional File 4F. Coach.** Numbers in parentheses for each behavioral change technique are per BCTTv1. *BCTTv1* BCT Taxonomy version 1 (Michie et. al., 2013).
